# Lipid Level, Lipid Variability, and Risk of Multiple Myeloma: A Nationwide Population-Based Study of 3,527,776 Subjects

**DOI:** 10.3390/cancers13030540

**Published:** 2021-01-31

**Authors:** Taewoong Choi, In Young Choi, Kyungdo Han, Su-Min Jeong, Jung Eun Yoo, Sang Youl Rhee, Yong-Gyu Park, Dong Wook Shin

**Affiliations:** 1 Division of Hematologic Malignancies and Cellular Therapy, Duke University Medical Center, Durham, NC 27710, USA; teawoong.choi@duke.edu; 2 Total Healthcare Center, Kangbuk Samsung Hospital, Sungkyunkwan University School of Medicine, Seoul 04514, Korea; inyoungb.choi@samsung.com; 3 Department of Statistics and Actuarial Science, Soongsil University, Seoul 06978, Korea; 4 Department of Family Medicine, Seoul Metropolitan Government-Seoul National University Boramae Medical Center, Seoul 07061, Korea; dpsme@sun.ac.kr; 5 Department of Nutrition, Harvard T.H. Chan School of Public Health, Boston, MA 02115, USA; 6 Department of Family Medicine, Healthcare System Gangnam Center, Seoul National University Hospital, Seoul 06236, Korea; 83259@snuh.org; 7 Department of Endocrinology and Metabolism, Kyung Hee University School of Medicine, Seoul 02453, Korea; rheesy@khu.ac.kr; 8 Department of Medical Statistics, College of Medicine, Catholic University of Korea, Seoul 06591, Korea; ygpark@catholic.ac.kr; 9 Supportive Care Center/Department of Family Medicine, Samsung Medical Center, Sungkyunkwan University School of Medicine, Seoul 06351, Korea; 10 Department of Clinical Research Design & Evaluation, Samsung Advanced Institute for Health Science & Technology (SAIHST), Sungkyunkwan University, Seoul 06355, Korea; 11 Department of Digital Health, Samsung Advanced Institute for Health Science & Technology (SAIHST), Sungkyunkwan University, Seoul 06355, Korea

**Keywords:** lipid level, lipid variability, risk, multiple myeloma

## Abstract

**Simple Summary:**

There is preclinical evidence that abnormalities in lipid metabolism promote cancer development, and a few studies show the association between lipid levels and multiple myeloma (MM). However, to our knowledge, the role of lipid variability as a risk factor for MM has not been evaluated. We investigated whether lipid level and its variability are associated with the development of MM at a population level. Lower baseline lipid levels of total cholesterol, high-density lipoprotein cholesterol, low-density lipoprotein cholesterol and triglycerides, and high variability in high-density lipoprotein cholesterol were all associated with increased risk of developing MM. These findings support the role of lipid metabolism in MM risk.

**Abstract:**

(1) Background: There is evidence that abnormality in lipid metabolism promotes cancer development. This study investigated whether lipid level and its variability are associated with the development of MM at a population level. (2) Methods: A retrospective cohort study included a total of 3,527,776 subjects aged 40 and above who participated in ≥3 health examinations within the previous five years, including the index year (2012–2013). Total cholesterol (TC), high-density lipoprotein cholesterol (HDL-C), low-density lipoprotein cholesterol (LDL-C) and triglyceride (TG) were measured, and visit-to-visit lipid variability were calculated by variability independent of the mean (VIM) method. The study population was followed from the health examination date in the index year until the diagnosis of MM, death, or the last follow-up date (31 December 2017). (3) Results: During a median (5–95%) 5.1 years of follow-up, 969 subjects developed MM. A lower risk of MM was observed with higher quartiles of baseline lipid levels compared to the lowest quartile group (Q4 vs. Q1: adjusted hazard ratios (aHRs) 0.51, 95% confidence interval (CI) (0.42–0.61) for TC; 0.50 (0.41–0.61) for HDL-C; 0.65 (0.54–0.77) for LDL-C; and 0.72 (0.60–0.87) for TG in model (3). Among all lipid measures, only variability in HDL-C was associated with risk of MM: aHRs (95% CI) were 1.12 (0.91–1.38), 1.19 (0.97–1.46), and 1.34 (1.09–1.65) in the Q2, Q3, and Q4, respectively, compared to the Q1 of VIM of HDL-C. (4) Conclusions: This study shows that patients with lower lipid levels and high HDL-C variability are at increased risk of developing MM.

## 1. Background

Multiple myeloma (MM), one of the most common blood cancers, originates from plasma cells. In the United States, there are about 32,000 newly diagnosed cases of MM every year [1]. Although MM survival outcomes have improved dramatically [2,3], MM is still an incurable disease that requires life-long treatment. It is well known that MM progresses from (monoclonal gammopathy of undetermined significance (MGUS), but the detailed pathogenesis remains elusive.

Lipids play an important role in cell growth and proliferation, and there is preclinical evidence that abnormality in lipid metabolism promotes cancer development, invasion, and metastasis via multiple signaling pathways [4,5,6,7]. In addition, epidemiological studies suggested serum lipid level is associated with future cancer risk, including breast [8,9,10,11], prostate [8,12], colon [8,13], and lung cancer [14]. As a result, lipid metabolism has emerged as a novel target for cancer prevention and treatment [4].

Data on lipid metabolism and hematologic malignancy, including MM, are still scarce. Several studies have shown that the levels of low-density lipoprotein cholesterol (LDL-C), high-density lipoprotein cholesterol (HDL-C) and total cholesterol (TC) were significantly lower in patients with MM [15,16,17]. However, only a handful of studies have investigated the association between lipid profile and future cancer risk. A Women’s Health Initiative study involving 24 thousand women showed a borderline inverse association between HDL-C and MM (adjusted hazard ratio (aHR), 95% confidence interval (95% CI) for highest to lowest quartile = 0.56, 0.31–1.01) [18] and a recent Danish study involving 117 thousand people reported that low levels of HDL-C cholesterol were significantly associated with increased risk of MM (aHR (95% CI) for one standard deviation (SD) decrease in HDL-C = 1.73 (1.28–2.35)) [19]. Recently, our previous study showed that low HDL was associated with higher hematological cancer risk, including MM (aHR, 95% CI for lowest to highest quartile = 1.63, 1.48–1.78) [20].

In addition, lipid level fluctuates substantially over time, even on a day-to-day basis [21,22]. Currently, lipid variability is regarded as a distinct feature apart from the lipid level itself [23]. Recent epidemiologic data suggest that visit-to-visit variability in lipid levels is associated with various health outcomes, such as coronary heart disease [24,25,26,27], stroke [24,27], end-stage renal disease [28], and mortality [26,27]. However, to our knowledge, the role of lipid variability as a risk factor for MM has not been evaluated, including our previous study regarding lipid levels and hematological cancer risk [20].

To better understand the association between lipid level and lipid variability and the risk of MM, large-scale, nationally representative data from the Korean National Health Insurance System (NHIS) were analyzed.

## 2. Methods

### 2.1. Data Source

The Korean government has a single mandatory health insurance system that covers nearly 97% of South Koreans, and the remaining 3% are covered by the Medical Aid program. NHIS manages all administrative processes and reimburses medical providers and pharmacies based on their claims for provision of medical and pharmacy services. NHIS also provides biennial health examinations for all Korean people aged 40 or older. The health examinations consist of anthropometric measurements, laboratory tests (lipid profiles, blood glucose, etc.) and questionnaires regarding lifestyle behaviors (smoking, alcohol consumption, and physical activity).

The Korean NHIS database contains the health information of all Korean people (~50 million), including eligibility (age, sex, place of residence, income level, etc.), medical utilization (diagnosis code (International Classification of Diseases [ICD] 10th Revision), diagnostic and therapeutic procedures, prescriptions, medical expenses), and results of health examinations [29]. These data are available from https://nhiss.nhis.or.kr/bd/ay/bdaya001iv.do with the permission of the NHIS and have been widely used for various epidemiologic studies [30,31].

### 2.2. Study Design and Ethics Statement

This retrospective cohort study using the NHIS database was approved by the Institutional Review Board of Samsung Medical Center (SMC 2018-08-112). Anonymized and de-identified information were used for analyses. Therefore, informed consent was not obtained. The database is open to all researchers with study protocols approved by the official review committee.

### 2.3. Study Population

Subjects included in the current study were those who participated in the health screening examinations in 2012 or 2013 (index year, considered as baseline) and had three or more health examinations within the previous four years including the index year. Of 19,459,018 subjects with health screening data in the index year, 5,632,394 participated in three or more health screening examinations during the previous four years including the index date. We excluded 1,979,276 subjects younger than 40 years old and 89,357 subjects with any cancer diagnosis before the index date. Subjects with missing variables (*n* = 35,985) were also excluded. Ultimately, the study population consisted of 3,527,776 subjects.

### 2.4. Data Collection and Measurements

Blood samples were collected on the health screening day after at least 8 h of fasting. Samples were analyzed for blood glucose and lipid profile including total cholesterol (TC), triglyceride (TG), and high-density lipoprotein cholesterol (HDL-C). Low-density lipoprotein cholesterol (LDL-C) level was calculated by the Friedewald Equation (LDL-C (mg/dL) = TC – HDL-C − (TG/5)) when TG level was less than 400 mg/dL. Otherwise, LDL-C level was measured by direct assay. Medical institutions and laboratories providing health screenings were certified by the NHIS via regular quality checks. Information on health behaviors was obtained by questionnaire.

### 2.5. Definition of Lipid Variability

Lipid variability was defined as the variation in values of each lipid profile between health screenings. Three indices of variability were used: Variability Independent of the Mean (VIM), Coefficient of Variation (CV), and Average Real Variability (ARV). VIM was initially used to minimize the correlation between the measurement of variability and the mean value. VIM was defined as 100 × standard deviation (SD)/mean^β^, where β is the regression coefficient using the natural logarithm of SD divided by the natural logarithm of the mean. CV and ARV are calculated according to the following formulas:

CV = (SD/mean lipid levels) × 100

$$\mathrm{ARV}=\frac{1}{N\;-\;1}\overset{n\;-\;1}{\underset{k\;=\;1}{\sum }}|\;{\mathrm{Lipid}\;}_{K\;+\;1}\;-\;{\mathrm{Lipid}\;}_{k}\;|$$

VIM was used for primary analysis, and CV and ARV were used for sensitivity analysis [23].

### 2.6. Definition of Covariates

Information on smoking (non-, ex-, and current smoker) and alcohol consumption (no, mild, and heavy drinking) was categorized into three groups. Physical activity was dichotomized by regularity, which was defined as strenuous physical activity performed for ≥20 min more than once a week. Body mass index (BMI, kg/m^2^) was calculated based on measured height and weight on health examination day and was treated as a continuous variable.

Comorbidities were defined using diagnosis codes of the International classification of disease version 10 (ICD 10), prescription information, and health screening results as follows: hypertension (I10-11 claim codes plus ≥1 prescription of an antihypertensive agent, or systolic/diastolic BP ≥ 140/90 mmHg); diabetes (E10–14 claim codes plus ≥1 prescription of an antidiabetic medication, or fasting glucose level ≥ 126 mg/dL); dyslipidemia (E78 claim code plus ≥1 prescription of lipid-lowering agent, or TC ≥ 240 mg/dL).

### 2.7. Study Outcomes and Follow-up

The incidence of MM was defined by new claims for inpatient or outpatient care with diagnosis codes for MM (C90.0) with registration in the special co-payment reduction program for critical illnesses. In Korea, all patients with cancer diagnoses can apply for reduced copayment (5% of medical bills, compared to 20 to 30% for general diseases). This application requires a medical certificate issued by a treating physician, and most people apply for it to reduce the out-of-pocket cost. This information was used to verify the cancer diagnosis in studies using the NHIS database [32].

The study population was followed from the index date to the date of diagnosis of MM, death, or until 31 December 2017, whichever came first.

### 2.8. Data Analyses

Cox proportional hazards regression modeling was used to estimate the hazard ratios (HRs) for MM associated with lipid level and lipid variability. The proportional hazards assumption was verified by evaluating parallelism between the curves of the log–log survivor function for different categories of variables and the Schoenfeld residual plots for quartile groups of lipid level and lipid variabilities.

Lipid level and variability were categorized into quartiles (Q1, Q2, Q3, and Q4) of VIM, CV, and ARV for each lipid level (TC, HDL-C, LDL-C, and TG). The lowest quartile (Q1) as a reference was compared with the other groups of quartiles (Q2, Q3, and Q4). Multivariate models were adjusted for age and sex (model 1) + smoking, alcohol consumption, physical activity [33], BMI [34], and diabetes (model 2) + lipid-lowering medications (model 3) + baseline levels of lipid profiles (TC, LDL-C, HDL-C, TG) (model 4, for lipid variability analyses). Because variability in HDL-C was the most predictive of incident MM, Table 1 displays detailed results according to HDL-C variability. Other indices of variability, such as CV and ARV, and 1-year lag time were used for sensitivity analyses. All analyses were performed using SAS version 9.4 (SAS Institute Inc., Cary, NC, USA), and a *p*-value < 0.05 was considered statistically significant.

## 3. Results

### 3.1. Baseline Characteristics of Study Population

The characteristics of participants classified by quartiles of VIM of HDL-C are described in Table 1. The mean HDL-C levels (mg/dL) (SD) were 62.0 (13.2), 56.8 (12.5), 52.6 (14.0), and 47.0 (16.0) in the first, second, third, and fourth quartiles, respectively. Subjects in higher quartiles of HDL-C variability were older, more likely to be men and current smokers, had higher BMI, and had a higher prevalence of comorbid conditions, such as diabetes and hypertension.

### 3.2. Lipid Levels and Risk of MM

During a median (5–95%) 5.1 (5.0–5.6) years of follow-up after the lipid variability assessment period, 969 (5.4 cases per 100,000 person-years) subjects developed MM.

**Figure 1:**
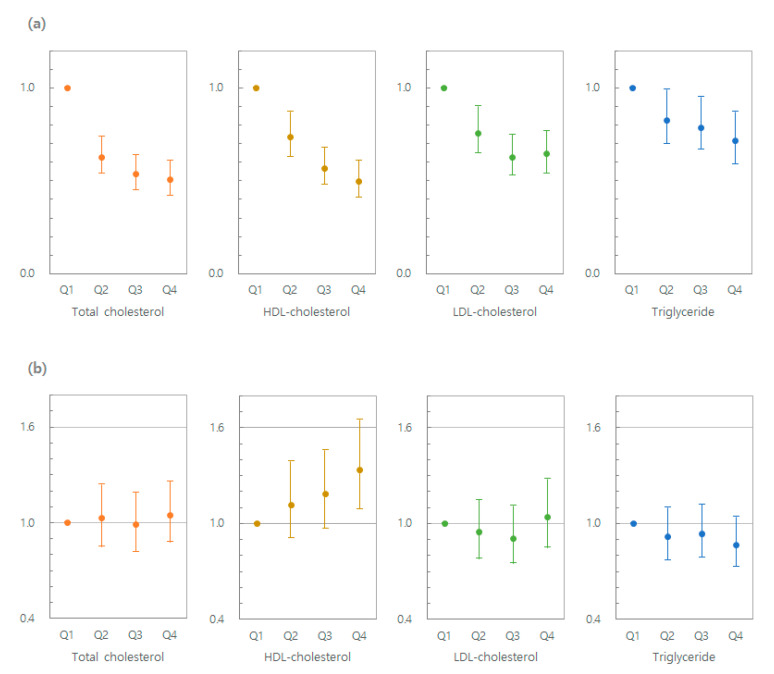
(**a**) Risk of multiple myeloma by quartile for lipid levels at baseline (Model 3) adjusted for age, sex, body mass index, smoking, alcohol consumption, physical activity, diabetes, and lipid-lowering medication. (**b**) Risk of multiple myeloma by quartile for lipid variability (Model 4) adjusted for age, sex, body mass index, smoking, alcohol consumption, physical activity, diabetes, lipid-lowering medication, and baseline lipid levels (TC, HDL-C, LDL-C, TG).

Table 2 and Figure 1a shows HRs for MM according to baseline lipid levels (TC, HDL-C, LDL-C, and TG). A lower risk of MM was observed with higher quartiles of baseline lipid levels compared to the lowest quartile group (Q4 vs. Q1: adjusted hazard ratio (aHR) 0.51 (95% confidence interval (CI) 0.42–0.61] for TC; 0.50 (0.41–0.61) for HDL-C; 0.65 (0.54–0.77) for LDL-C; and 0.72 (0.60–0.87) for TG in model 3).

### 3.3. Lipid Variability and Risk of MM

Table 3 and Figure 1b presents associations between indices of lipid variability and risk of MM. An incrementally higher risk of MM was observed for higher VIM quartiles (Q2–Q4) compared with the lowest quartile VIM group (Q1) in all models. After adjusting for age, sex, BMI, smoking, alcohol consumption, regular exercise, diabetes, lipid-lowering medication, and baseline HDL-C levels, aHRs (95% CI) for incident MM were 1.12 (0.91–1.38), 1.19 (0.97–1.46), and 1.34 (1.09–1.65) in the second (Q2), third (Q3), and fourth quartiles (Q4), respectively, compared to the first quartile (Q1) of VIM of HDL-C. In contrast to HDL-C variability, the variability in TC, LDL-C, and TG was not significantly associated with risk of MM.

In sensitivity analyses with other variability parameters, higher HDL-C variability assessed by CV and ARV was again significantly associated with increased risk of MM compared to the lowest quartile of CV and ARV. Variability of other lipid levels (TC, LDL-C, and TG) again did not show significant association with incident MM. (Tables S1 and S2). In sensitivity analysis with one-year lag time, the association of lipid level and HDL-C variability with the risk of MM was consistent with the main analysis (Tables S3 and S4).

## 4. Discussion

To our knowledge, this is the first study demonstrating that lower lipid level and higher visit-to-visit variability in HDL-C levels are associated with increased risk of MM, irrespective of lipid levels. The strengths of this study include (1) a large, population-based database linked to a claims database, enabling investigation of relatively rare clinical outcomes such as MM, (2) near-complete follow-up, and (3) robust results across various sensitivity and stratified analyses.

This study clearly confirmed inverse association between lipid level and the risk of MM consistent with previous studies [18,19,20]. The magnitude of association was also similar at 1.5~2.0 times risk when comparing lowest to highest quartile of HDL-C. Although the underlying mechanisms are not well understood, there are several potential explanations on why low lipid levels, especially low HDL-C level, is associated with increased risk of MM.

First, HDL-C itself could have a protective role against MM through antioxidant and anti-inflammatory properties [35]. In hematological malignancies, there is a crosstalk between low-grade chronic inflammation, dyslipidemia, and oxidative stress, as evidenced in non-Hodgkin’s lymphoma [36]. Inflammatory pathways activated by immune factors and genetic alterations affecting oncogenes are part of the mechanisms leading to carcinogenesis [37]. HDL-C suppresses myeloid proliferation and leukocytosis by decreasing granulocyte-monocyte progenitors and proliferation of interleukin-3 in bone marrow cells [38,39]. Apolipoprotein A-I (Apo A-I), a major protein constituent of HDL-C, also has protective roles against cancer development through anti-inflammatory activities [40]. Reduced Apo A-I levels were observed at the time of acute lymphoblastic leukemia diagnosis in children [41].

Conversely, reduced HDL-C levels might be a secondary phenomenon driven by cancer cell metabolism. Malignant cells can induce liposynthesis and accumulate intracellular cholesteryl esters for new membrane biogenesis [42]. Scavenger receptor class B type (SR-BI), an HDL-C receptor, is highly expressed on tumor cell surfaces. SR-BI facilitates uptake of cholesteryl esters from HDL-C into cytoplasm, leading to a significant reduction in plasma HDL-C levels [43]. The (mammalian target of rapamycin (mTOR) pathway is known to be activated in multiple myeloma and may play an important role in this process by potentially up-regulating the scavenger receptor (SR-BI), although detailed mechanism information is still elusive [44,45,46,47]. In the same context, preclinical studies showed low cholesterol level in the culture medium was related to neoplastic cellular growth, suggesting that cholesterol is used by lymphoma cells for their progression [48,49]. In addition, some clinical studies suggested that low cholesterol levels can be accompanied by hematologic malignancies such as MM and chronic lymphocytic leukemia [15,16,17,50], and other studies showed that reduced HDL-C levels returned to normal after successful treatment of leukemia and lymphoma [51,52], suggesting that HDL-C could potentially serve as a biomarker of tumor burden.

The novel finding of our study is that even after adjustment of baseline HDL-C level, variability of HDL-C was independently associated with further increased risk of MM. Again, mechanisms linking higher HDL-C variability and increased risk of MM development remain unclear. The first possibility is that there are common factors cause both higher HDL-C variability and MM development without a causal relationship. High lipid variability was commonly observed in patients with hypertension, diabetes mellitus, dyslipidemia, and cardiovascular disease. Recently, cardiovascular disease and cancer were shown to share a common element of inflammation. Another possibility is that HDL-C variability may potentially promote the development of MM. As cholesterol can influence gene expression in cancer cells, it is plausible that variation in cholesterol level can directly contributes to the development of MM. The last remaining possibility is reverse causality, which means that undiagnosed MM can increase fluctuation in HDL-C levels, as noted in above explanations on altered lipid metabolism by tumor burden.

The clinical significance of this study result remains to be determined, as current understanding of the detailed relationship between lipid metabolism and the development of MM is limited. Low lipid level and high HDL-C variability can be a novel modifiable risk factor or secondary to MM development. Although it still remains uncovered whether lipid variability truly reflects a reproducible phenomenon and whether monitoring lipid fluctuation over a shorter duration of time (daily or weekly) is useful for clinical risk assessment, the association demonstrated in this study may provide a clue for future research. Recently, there is growing evidence that statin use is effective in preventing MM [53,54,55]. As statin can reduce both lipid level and lipid variability, our results might support such observations.

Currently, dyslipidemia is managed mainly for the prevention of cardiovascular disease. In general, lower TC, TG, LDL and higher HDL is regarded as cardio-protective. However, our study showed higher levels of all four lipid parameters are associated with lower MM risk. It is probable that we should consider cancer risk in the management of dyslipidemia in the future. MM risk according to the common reference range used for cardiovascular disease is shown in Table S5.

This study has some limitations. First, causality cannot be determined because of the observational nature of this study. However, sensitivity analyses with a 1-year lag period showed similar results. Second, as our study is based on routinely collected data from numerous medical institutions, lipid measurement could not be as standardized as in other prospective cohort studies utilizing central laboratories. However, there is a certification process for quality control implemented by the NHIS and a large sample size would mitigate such an effect. Third, our study only included a South Korean population. The prevalence of dyslipidemia varies between ethnic groups [56,57], and intra-individual lipid variability may be influenced by genetic and environmental factors [23]; therefore, generalization to other ethnic groups should be established through further studies in other populations. Lastly, considering asymptomatic precursor states preceding MM, the follow-up time considered in this study was relatively short.

## 5. Conclusions

In conclusion, this study clearly demonstrates that serum lipid levels and HDL-C variability are associated with MM risk using a nationwide population-based cohort. This study may provide a clue for the role of lipid metabolism in the risk of MM. Future studies should explore the details of a biological mechanism linking lipid level, HDL-C variability, and MM development.

## Figures and Tables

**Table 1:** Baseline characteristics of study population according to variability independent of the mean (VIM) of high density lipoprotein in quartiles.

	Variability Independent of Mean (VIM) of HDL				
Characteristics	Q1	Q2	Q3	Q4	*p*
N	881,919	881,952	881,989	881,916	
Age, years	51.2 ± 8.6	51.5 ± 8.7	52.0 ± 8.9	53.1 ± 9.5	<0.0001
Sex (Male)	483,552 (54.8)	544,238 (61.7)	587,485 (66.6)	635,738 (72.1)	<0.0001
Lipid profiles (mg/dL)					
TC	201.3 ± 34.6	199.2 ± 35.2	197.2 ± 35.8	193.0 ± 37.0	<0.0001
HDL-C	62.0 ± 13.2	56.8 ± 12.5	52.6 ± 14.0	47.0 ± 16.0	<0.0001
LDL-C	116.8 ± 33.2	117.5 ± 34.2	117.22 ± 35.08	114.47 ± 36.95	<0.0001
TG *	99.8 (99.7–99.9)	110.4 (110.2–110.5)	121.5 (121.4–121.7)	141.1 (140.9–141.2)	<0.0001
Living place (Urban)	407,633 (46.2)	400,125 (45.4)	393,781 (44.7)	378,010 (42.9)	<0.0001
Household income (Low)	195,558 (22.2)	190,524 (21.6)	192,546 (21.8)	201,809 (22.9)	<0.0001
BMI, kg/m^2^	23.5 ± 3.0	23.8 ± 3.0	24.1 ± 2.9	24.5 ± 2.9	<0.0001
WC, cm	79.3 ± 8.6	80.7 ± 8.5	81.8 ± 8.3	83.4 ± 8.2	<0.0001
Smoking habits					<0.0001
No	527,768 (59.8)	483,106 (54.8)	451,638 (51.2)	415,426 (47.1)	
Ex	168,446 (19.1)	185,731 (21.1)	194,658 (22.1)	198,829 (22.6)	
Current	185,705 (21.1)	213,115 (24.2)	235,693 (26.7)	267,661 (30.4)	
Alcohol consumption					<0.0001
No	416,162 (47.2)	408,334 (46.3)	412,138 (46.7)	435,252 (49.4)	
Mild (<30 mg/d)	401,658 (45.5)	406,167 (46.1)	402,049 (45.6)	381,322 (43.2)	
Heavy (≥30 mg/d)	64,099 (7.3)	67,451 (7.7)	67,802 (7.7)	65,342 (7.4)	
Regular exercise, Yes	205,121 (23.3)	205,180 (23.3)	203,589 (23.1)	197,258 (22.4)	<0.0001
Hypertension, Yes	226,922 (25.7)	245,354 (27.8)	266,667 (30.2)	303,871 (34.5)	<0.0001
Diabetes, Yes	69,405 (7.9)	82,132 (9.3)	97,362 (11.0)	126,127 (14.3)	<0.0001
Dyslipidemia, Yes	210642 (23.9)	207,056 (23.5)	203,745 (23.1)	196,157 (22.2)	<0.0001
Blood glucose, mg/dL	97.7 ± 20.7	98.8 ± 22.1	99.9 ± 23.6	101.9 ± 26.3	<0.0001
SBP, mmHg	121.9 ± 14.3	122.7 ± 14.2	123.3 ± 14.1	124.2 ± 14.1	<0.0001
DBP, mmHg	76.4 ± 9.8	76.9 ± 9.7	77.4 ± 9.7	77.8 ± 9.7	<0.0001

Abbreviation: TC, total cholesterol; HDL-C, high-density lipoprotein cholesterol; LDL-C, low-density lipoprotein cholesterol; TG, triglyceride; BMI, body mass index; WC, waist circumference; SBP, systolic blood pressure; DBP, diastolic blood pressure. * geometric means (95% confidence interval). Continuous and categorical variables were presented as the mean ± standard deviation and number (%), respectively.

**Table 2:** Risk of Multiple Myeloma by quartiles of lipid levels at baseline.

Lipid Levels	N	Case	Duration	IR (100,000 PY)	HR (95% CI)		
					Model 1	Model 2	Model 3
TC							
Q1	867,617	377	4,449,425.7	8.5	1 (ref.)	1 (ref.)	1 (ref.)
Q2	905,931	234	4,654,278.6	5.0	0.63 (0.54, 0.74)	0.63 (0.54, 0.74)	0.63 (0.54, 0.74)
Q3	885,382	190	4,550,159.6	4.2	0.54 (0.45, 0.64)	0.54 (0.45, 0.64)	0.54 (0.45, 0.64)
Q4	868,846	168	4,455,274.4	3.8	0.51 (0.42, 0.61)	0.51 (0.42, 0.61)	0.51 (0.42, 0.61)
HDL-C							
Q1	846,909	362	4,350,341.4	8.3	1 (ref.)	1 (ref.)	1 (ref.)
Q2	873,920	255	4,493,565.9	5.7	0.73 (0.63, 0.86)	0.74 (0.63, 0.87)	0.74 (0.63, 0.87)
Q3	921,806	197	4,737,000.3	4.2	0.56 (0.47, 0.67)	0.57 (0.48, 0.68)	0.57 (0.48, 0.68)
Q4	885,141	155	4,528,230.7	3.4	0.49 (0.41, 0.59)	0.51 (0.41, 0.62)	0.50 (0.41, 0.61)
LDL-C							
Q1	890,525	326	4,561,655.5	7.1	1 (ref.)	1 (ref.)	1 (ref.)
Q2	861,343	240	4,424,092.2	5.4	0.77 (0.65, 0.91)	0.76 (0.65, 0.90)	0.76 (0.65, 0.90)
Q3	888,661	202	4,567,970.3	4.4	0.64 (0.54, 0.76)	0.63 (0.53, 0.75)	0.63 (0.53, 0.75)
Q4	887,247	201	4,555,420.3	4.4	0.66 (0.55, 0.79)	0.65 (0.54, 0.77)	0.65 (0.54, 0.77)
TG							
Q1	889,167	268	4,558,628.0	5.9	1 (ref.)	1 (ref.)	1 (ref.)
Q2	880,387	248	4,517,069.2	5.5	0.85 (0.71, 1.01)	0.83 (0.70, 0.99)	0.83 (0.70, 0.99)
Q3	877,719	245	4,508,015.7	5.4	0.82 (0.69, 0.98)	0.80 (0.67, 0.95)	0.79 (0.67, 0.95)
Q4	880,503	208	4,525,425.4	4.6	0.75 (0.63, 0.90)	0.72 (0.60, 0.87)	0.72 (0.59, 0.87)

Abbreviation: TC, total cholesterol; HDL-C, high-density lipoprotein cholesterol; LDL-C, low-density lipoprotein cholesterol; TG, triglyceride; IR, incidence rate; PY, person-years; HR, hazard ratio; CI, confidence interval. Model 1 was adjusted for age and sex. Model 2 was adjusted for age, sex, body mass index, smoking, alcohol consumption, physical activity, and diabetes. Model 3 was adjusted for age, sex, body mass index, smoking, alcohol consumption, physical activity, diabetes, and lipid-lowering medication.

**Table 3:** Risk of Multiple Myeloma by quartiles of lipid variability (VIM).

VIM	N	Case	Duration	IR (100,000 PY)	HR (95% C.I.)			
					Model 1	Model 2	Model 3	Model 4
TC								
Q1	881,943	223	4,511,433.7	4.9	1 (ref.)	1 (ref.)	1 (ref.)	1 (ref.)
Q2	881,943	230	4,546,886.3	5.1	1.04 (0.86, 1.25)	1.04 (0.86, 1.25)	1.04 (0.86, 1.25)	1.03 (0.85, 1.24)
Q3	881,945	231	4,543,898.0	5.1	1.02 (0.85, 1.23)	1.02 (0.85, 1.23)	1.02 (0.85, 1.23)	0.99 (0.82, 1.19)
Q4	881,945	285	4,506,920.3	6.3	1.16 (0.98, 1.38)	1.16 (0.97, 1.38)	1.16 (0.97, 1.38)	1.05 (0.88, 1.26)
HDL-C								
Q1	881,919	158	4,508,215.7	3.5	1 (ref.)	1 (ref.)	1 (ref.)	1 (ref.)
Q2	881,952	203	4,538,730.6	4.5	1.22 (0.99, 1.51)	1.21 (0.99, 1.49)	1.21 (0.99, 1.49)	1.12 (0.91, 1.39)
Q3	881,989	249	4,540,299.4	5.5	1.40 (1.15, 1.71)	1.38 (1.13, 1.68)	1.38 (1.13, 1.68)	1.19 (0.97, 1.46)
Q4	881,916	359	4,521,892.8	7.9	1.77 (1.47, 2.14)	1.72 (1.43, 2.09)	1.73 (1.43, 2.09)	1.34 (1.09, 1.65)
LDL-C								
Q1	881,936	212	4,516,264.2	4.7	1 (ref.)	1 (ref.)	1 (ref.)	1 (ref.)
Q2	881,952	218	4,541,908.7	4.8	1.02 (0.84, 1.23)	1.02 (0.84, 1.23)	1.02 (0.84, 1.23)	0.95 (0.78, 1.15)
Q3	881,942	230	4,535,323.7	5.1	1.05 (0.87, 1.27)	1.05 (0.87, 1.27)	1.05 (0.87, 1.27)	0.91 (0.75, 1.11)
Q4	881,946	309	4,515,641.8	6.8	1.34 (1.12, 1.59)	1.35 (1.13, 1.61)	1.35 (1.13, 1.61)	1.04 (0.85, 1.28)
TG								
Q1	881,944	265	4,499,237.3	5.9	1 (ref.)	1 (ref.)	1 (ref.)	1 (ref.)
Q2	881,944	239	4,540,799.6	5.3	0.92 (0.78, 1.10)	0.93 (0.78, 1.10)	0.93 (0.78, 1.10)	0.92 (0.77, 1.10)
Q3	881,944	242	4,545,779.6	5.3	0.94 (0.79, 1.12)	0.95 (0.79, 1.13)	0.95 (0.79, 1.13)	0.94 (0.79, 1.12)
Q4	881,944	223	4,523,321.9	4.9	0.88 (0.73, 1.05)	0.89 (0.74, 1.06)	0.89 (0.74, 1.06)	0.87 (0.73, 1.04)

Abbreviation: VIM, variability independent of the mean; TC, total cholesterol; HDL-C, high-density lipoprotein cholesterol; LDL-C, low-density lipoprotein cholesterol; TG, triglyceride; IR, incidence rate; PY, person-years; HR, hazard ratio; CI, confidence interval. Model 1 was adjusted for age and sex. Model 2 was adjusted for age, sex, body mass index, smoking, alcohol consumption, physical activity, and diabetes. Model 3 was adjusted for age, sex, body mass index, smoking, alcohol consumption, physical activity, diabetes, and lipid-lowering medication. Model 4 was adjusted for age, sex, body mass index, smoking, alcohol consumption, physical activity, diabetes, lipid-lowering medication, and baseline lipid levels (TC, HDL-C, LDL-C, TG).

## Data Availability

Restrictions apply to the availability of these data. Data were obtained from Korea NHIS and are available at https://nhiss.nhis.or.kr/bd/ay/bdaya001iv.do with the permission of Korea NHIS.

## Supplementary Materials

The following are available online at https://www.mdpi.com/2072-6694/13/3/540/s1, Table S1: Hazard ratios and 95% confidence intervals of multiple myeloma by quartiles of lipid variability: sensitivity analysis with coefficient of variation (CV), Table S2: Hazard ratios and 95% confidence intervals of multiple myeloma by quartiles of lipid variability: sensitivity analysis with average real variability (ARV), Table S3: Risk of multiple myeloma by quartiles of lipid levels at baseline: sensitivity analysis with 1-year lag time, Table S4: Hazard ratios and 95% confidence intervals of multiple myeloma by quartiles of lipid variability: sensitivity analysis with 1-year lag time, Table S5: Risk of multiple myeloma by lipid level at baseline, Table S6: STROBE checklist.

## Abbreviation

ARV	Average Real Variability
BMI	Body mass index
CV	Coefficient of Variation
HR	Hazard ratio
HDL-C	High-density lipoprotein cholesterol
ICD	International Classification of Diseases
LDL-C	Low-density lipoprotein cholesterol
MM	Multiple myeloma
NHIS	National Health Insurance System
Q	Quartile
SD	Standard deviation
TC	Total cholesterol
TG	Triglyceride
VIM	Variability independent of the mean
