# Supplementary material for: Lipid Level, Lipid Variability, and Risk of Multiple Myeloma: A Nationwide Population-Based Study of 3,527,776 Subjects
Source: Cancers (Basel). 2021 Jan 31;13(3):540. doi: 10.3390/cancers13030540 (PMC7866996; doi:10.3390/cancers13030540)
Supplement: Supplementary file 1 [file cancers-13-00540-s001.pdf]

Supplementary Materials

Lipid Level, Lipid Variability, and Risk of Multiple Myeloma: A Nationwide Population-Based Study of 3,527,776 Subjects

Taewoong Choi, In Young Choi, Kyungdo Han, Su-Min Jeong, Jung Eun Yoo, Sang Youl Rhee, Yong Gyu Park and Dong Wook Shin

Table S1. Hazard ratios and 95% confidence intervals of multiple myeloma by quartiles of lipid variability: sensitivity analysis with coefficient of variation (CV).

CV	N	Case	Duration	IR (100,000 PY)	HR (95% C.I.)			
					Model 1	Model 2	Model 3	Model 4
**TC**								
Q1	881,945	223	4,511,322.3	4.9	1 (ref.)	1 (ref.)	1 (ref.)	1 (ref.)
Q2	881,943	231	4,547,006.8	5.1	1.04 (0.87,1.25)	1.04 (0.87,1.25)	1.04 (0.87,1.25)	1.03 (0.86,1.24)
Q3	881,935	231	4,543,725.4	5.1	1.02 (0.85,1.23)	1.02 (0.85,1.23)	1.02 (0.85,1.23)	0.99 (0.82,1.19)
Q4	881,953	284	4,507,083.8	6.3	1.16 (0.97,1.38)	1.15 (0.97,1.37)	1.15 (0.97,1.38)	1.05 (0.88,1.26)
**HDL**								
Q1	881,850	174	4,513,302.0	3.9	1 (ref.)	1 (ref.)	1 (ref.)	1 (ref.)
Q2	882,276	238	4,548,454.4	5.2	1.35 (1.11,1.64)	1.35 (1.11,1.64)	1.35 (1.11,1.64)	1.35 (1.11,1.64)
Q3	881,712	244	4,538,126.9	5.4	1.32 (1.08,1.60)	1.32 (1.09,1.60)	1.32 (1.08,1.61)	1.31 (1.08,1.60)
Q4	881,938	313	4,509,255.1	6.9	1.49 (1.24,1.80)	1.50 (1.24,1.80)	1.50 (1.24,1.80)	1.47 (1.22,1.77)
**LDL**								
Q1	881,927	221	4,510,607.4	4.9	1 (ref.)	1 (ref.)	1 (ref.)	1 (ref.)
Q2	881,957	233	4,545,009.8	5.1	1.06 (0.88,1.27)	1.06 (0.88,1.27)	1.06 (0.88,1.27)	1.04 (0.86,1.25)
Q3	881,948	239	4,539,890.5	5.3	1.07 (0.89,1.28)	1.07 (0.89,1.28)	1.07 (0.89,1.28)	1.02 (0.85,1.22)
Q4	881,944	276	4,513,630.3	6.1	1.14 (0.96,1.36)	1.15 (0.96,1.37)	1.15 (0.96,1.37)	1.03 (0.86,1.23)
**TG**								
Q1	881,944	266	4,499,732.3	5.9	1 (ref.)	1 (ref.)	1 (ref.)	1 (ref.)
Q2	881,944	239	4,540,879.8	5.3	0.92 (0.77,1.10)	0.92 (0.77,1.10)	0.92 (0.77,1.10)	0.92 (0.77,1.09)
Q3	881,944	239	4,545,558.0	5.3	0.93 (0.78,1.11)	0.93 (0.78,1.11)	0.93 (0.78,1.11)	0.92 (0.77,1.10)
Q4	881,944	225	4,522,968.2	5.0	0.89 (0.74,1.06)	0.89 (0.75,1.07)	0.89 (0.75,1.07)	0.87 (0.73,1.04)

Abbreviation: CV, coefficient of variation; TC, total cholesterol; HDL, high-density lipoprotein cholesterol; LDL low-density lipoprotein cholesterol; TG, triglyceride; IR, incidence rate; PY, person-years; HR, hazard ratio; CI, confidence interval. Model 1 was adjusted for age and sex. Model 2 was adjusted for age, sex, body mass index, smoking, alcohol consumption, physical activity, and diabetes. Model 3 was adjusted for age, sex, body mass index, smoking, alcohol consumption, physical activity, diabetes, and lipid-lowering medication. Model 4 was adjusted for age, sex, body mass index, smoking, alcohol consumption, physical activity, diabetes, lipid-lowering medication, and baseline lipid levels (TC, HDL, LDL, TG).

Table S2. Hazard ratios and 95% confidence intervals of multiple myeloma by quartiles of lipid variability: sensitivity analysis with average real variability (ARV).

ARV	N	Case	Duration	IR (100000 PY)	HR (95% C.I.)			
					Model 1	Model 2	Model 3	Model 4
**TC**								
Q1	894,314	248	4,581,664.1	5.4	1 (ref.)	1 (ref.)	1 (ref.)	1 (ref.)
Q2	854,425	222	4,409,741.7	5.0	0.95 (0.79,1.13)	0.95 (0.79,1.13)	0.95 (0.79,1.13)	0.98 (0.82,1.17)
Q3	900,420	251	4,632,774.3	5.4	0.99 (0.84,1.19)	0.99 (0.84,1.19)	1.00 (0.84,1.19)	1.05 (0.88,1.25)
Q4	878,617	248	4,484,958.2	5.5	0.95 (0.80,1.13)	0.95 (0.79,1.13)	0.95 (0.79,1.13)	1.03 (0.86,1.23)
**HDL**								
Q1	912,762	210	4,695,851.5	4.5	1 (ref.)	1 (ref.)	1 (ref.)	1 (ref.)
Q2	823,541	233	4,239,822.6	5.5	1.21 (1.01,1.46)	1.22 (1.02,1.48)	1.22 (1.02,1.48)	1.27 (1.06,1.54)
Q3	919,488	273	4,721,358.7	5.8	1.23 (1.03,1.48)	1.26 (1.05,1.50)	1.26 (1.05,1.50)	1.36 (1.14,1.63)
Q4	871,985	253	4,452,105.5	5.7	1.12 (0.93,1.35)	1.15 (0.96,1.39)	1.15 (0.96,1.39)	1.34 (1.11,1.61)
**LDL**								
Q1	881,936	212	4,516,264.2	4.7	1 (ref.)	1 (ref.)	1 (ref.)	1 (ref.)
Q2	881,952	218	4,541,908.7	4.8	1.02 (0.84,1.23)	1.02 (0.84,1.23)	1.02 (0.84,1.23)	0.95 (0.78,1.15)
Q3	881,942	230	4,535,323.7	5.1	1.05 (0.87,1.27)	1.05 (0.87,1.27)	1.05 (0.87,1.27)	0.91 (0.75,1.11)
Q4	881,946	309	4,515,641.8	6.8	1.34 (1.12,1.59)	1.35 (1.13,1.61)	1.35 (1.13,1.61)	1.04 (0.85,1.28)
**TG**								
Q1	873,022	236	4,471,603.2	5.3	1 (ref.)	1 (ref.)	1 (ref.)	1 (ref.)
Q2	892,512	247	4,600,467.9	5.4	1.03 (0.86,1.23)	1.03 (0.86,1.23)	1.03 (0.86,1.23)	1.05 (0.88,1.25)
Q3	877,593	227	4,513,882.1	5.0	0.94 (0.78,1.13)	0.94 (0.78,1.13)	0.94 (0.78,1.13)	0.97 (0.82,1.16)
Q4	884,649	259	4,523,185.2	5.7	1.01 (0.84,1.20)	1.00 (0.84,1.20)	1.01 (0.84,1.20)	1.05 (0.88,1.25)

Abbreviation: ARV, average real variability; TC, total cholesterol; HDL, high-density lipoprotein cholesterol; LDL low-density lipoprotein cholesterol; TG, triglyceride; IR, incidence rate; PY, person-years; HR, hazard ratio; CI, confidence interval. Model 1 was adjusted for age and sex. Model 2 was adjusted for age, sex, body mass index, smoking, alcohol consumption, physical activity, and diabetes. Model 3 was adjusted for age, sex, body mass index, smoking, alcohol consumption, physical activity, diabetes, and lipid-lowering medication. Model 4 was adjusted for age, sex, body mass index, smoking, alcohol consumption, physical activity, diabetes, lipid-lowering medication, and baseline lipid levels (TC, HDL, LDL, TG).

Table S3. Risk of multiple myeloma by quartiles of lipid levels at baseline: sensitivity analysis with 1-year lag time.

Lipid Levels	N	Case	Duration	IR (100,000 PY)	HR (95% C.I.)		
					Model 1	Model 2	Model 3
**TC**							
Q1	866,130	326	3,582,323.9	9.1	1(ref.)	1(ref.)	1(ref.)
Q2	905,010	206	3,748,649.7	5.5	0.64 (0.54,0.77)	0.64 (0.54,0.77)	0.64 (0.54,0.77)
Q3	884,605	162	3,665,040.8	4.4	0.53 (0.44,0.64)	0.53 (0.44,0.64)	0.53 (0.44,0.64)
Q4	868,091	154	3,586,695.1	4.3	0.54 (0.45,0.66)	0.54 (0.45,0.66)	0.54 (0.44,0.66)
**HDL**							
Q1	845,599	299	3,503,897.1	8.5	1(ref.)	1(ref.)	1(ref.)
Q2	872,977	236	3,619,963.1	6.5	0.82 (0.70,0.98)	0.84 (0.71,1.00)	0.84 (0.71,1.00)
Q3	920,963	175	3,815,481.9	4.6	0.61 (0.50,0.73)	0.63 (0.52,0.76)	0.63 (0.52,0.76)
Q4	884,297	138	3,643,367.4	3.8	0.53 (0.44,0.66)	0.56 (0.46,0.70)	0.57 (0.46,0.70)
**LDL**							
Q1	889,110	283	3,671,607.9	7.7	1(ref.)	1(ref.)	1(ref.)
Q2	860,428	212	3,563,061.7	5.9	0.79 (0.66,0.94)	0.78 (0.65,0.93)	0.78 (0.65,0.93)
Q3	887,835	177	3,679,590.7	4.8	0.65 (0.54,0.78)	0.64 (0.53,0.77)	0.63 (0.3,0.77)
Q4	886,463	176	3,668,449.2	4.8	0.67 (0.55,0.81)	0.65 (0.54,0.79)	0.65 (0.54,0.79)
**TG**							
Q1	888,183	224	3,669,797.1	6.1	1(ref.)	1(ref.)	1(ref.)
Q2	879,341	221	3,637,047.8	6.1	0.90 (0.75,1.09)	0.88 (0.73,1.07)	0.88 (0.73,1.06)
Q3	876,702	217	3,630,640.6	6.0	0.87 (0.72,1.05)	0.84(0.69,1.01)	0.83 (0.69,1.01)
Q4	879,610	186	3,645,224.0	5.1	0.80 (0.66,0.98)	0.76 (0.62,0.94)	0.76 (0.62,0.93)

Abbreviation: TC, total cholesterol; HDL, high-density lipoprotein cholesterol; LDL low-density lipoprotein cholesterol; TG, triglyceride; IR, incidence rate; PY, person-years; HR, hazard ratio; CI, confidence interval. Model 1 was adjusted for age and sex. Model 2 was adjusted for age, sex, body mass index, smoking, alcohol consumption, physical activity, and diabetes. Model 3 was adjusted for age, sex, body mass index, smoking, alcohol consumption, physical activity, diabetes, and lipid-lowering medication.

Table S4. Hazard ratios and 95% confidence intervals of multiple myeloma by quartiles of lipid variability: sensitivity analysis with 1-year lag time.

HDL	N	Case	Duration	IR (100,000 PY)	HR (95% C.I.)			
					Model 1	Model 2	Model 3	Model 4
**VIM**								
Q1	881,245	137	3,626,508.7	3.8	1 (ref.)	1 (ref.)	1 (ref.)	1 (ref.)
Q2	881,171	189	3,657,049.8	5.2	1.31 (1.05,1.63)	1.29 (1.04,1.62)	1.29 (1.04,1.61)	1.22 (0.97,1.52)
Q3	881,043	219	3,658,643.0	5.9	1.41 (1.14,1.75)	1.38 (1.12,1.71)	1.38 (1.11,1.71)	1.23 (0.99,1.53)
Q4	880,377	303	3,640,507.9	8.3	1.72 (1.40,2.10)	1.65 (1.34,2.02)	1.64 (1.34,2.02)	1.34 (1.08,1.67)
**CV**								
Q1	881,122	157	3,631,691.6	4.3	1 (ref.)	1 (ref.)	1 (ref.)	1 (ref.)
Q2	881,501	215	3,666,439.6	5.8	1.35 (1.10,1.66)	1.35 (1.10,1.66)	1.35 (1.10,1.66)	1.35 (1.10,1.66)
Q3	880,760	211	3,656,753.1	5.8	1.26 (1.03,1.55)	1.26 (1.03,1.55)	1.26 (1.03,1.55)	1.26 (1.02,1.55)
Q4	880,453	265	3,627,825.2	7.3	1.40 (1.15,1.70)	1.40 (1.15,1.71)	1.40 (1.15,1.70)	1.38 (1.13,1.69)
**ARV**								
Q1	911,965	189	3,783,356.2	5.0	1 (ref.)	1 (ref.)	1 (ref.)	1 (ref.)
Q2	822,717	198	3,416,570.6	5.8	1.15 (0.94,1.40)	1.16 (0.95,1.41)	1.16 (0.95,1.41)	1.19 (0.98,1.46)
Q3	918,480	244	3,802,225.6	6.4	1.23 (1.01,1.48)	1.25 (1.03,1.51)	1.25 (1.03,1.51)	1.34 (1.11,1.62)
Q4	870,674	217	3,580,557.1	6.1	1.07 (0.88,1.30)	1.11 (0.91,1.35)	1.10 (0.91,1.34)	1.25 (1.03,1.53)

Abbreviation: VIM, variability independent of the mean; CV, coefficient of variation; ARV, average real variability; HDL, high-density lipoprotein cholesterol; IR, incidence rate; PY, person-years; HR, hazard ratio; CI, confidence interval. Model 1 was adjusted for age and sex. Model 2 was adjusted for age, sex, body mass index, smoking, alcohol consumption, physical activity, and diabetes. Model 3 was adjusted for age, sex, body mass index, smoking, alcohol consumption, physical activity, diabetes, and lipid-lowering medication. Model 4 was adjusted for age, sex, body mass index, smoking, alcohol consumption, physical activity, diabetes, lipid-lowering medication, and baseline lipid levels (TC, HDL, LDL, TG).

Table S5. Risk of Multiple Myeloma by cadiovascular categories of lipid levels at baseline.

Lipid Levels	N	Case	Duration	IR (100,000 PY)	HR (95% CI)		
					Model 1	Model 2	Model 3
**TC**							
<200	1,927,633	646	9,895,397.5	6.5	1 (ref.)	1 (ref.)	1 (ref.)
200–239	1,178,727	240	6,055,899.3	4.0	0.66 (0.57, 0.77)	0.67 (0.57, 0.77)	0.67 (0.57, 0.77)
≥240	421,416	83	2,157,841.6	3.8	0.65 (0.52, 0.82)	0.65 (0.51, 0.82)	0.65 (0.52, 0.82)
**HDL-C**							
<40	400,391	210	2,054,333.6	10.2	1 (ref.)	1 (ref.)	1 (ref.)
40–59	2,009,641	561	10,332,156.5	5.4	0.60 (0.51, 0.70)	0.61 (0.52, 0.72)	0.61 (0.52, 0.72)
≥60	1,117,744	198	5,722,648.2	3.5	0.42 (0.35, 0.51)	0.44 (0.35, 0.53)	0.43 (0.35, 0.53)
**LDL-C**							
<100	1,101,423	405	5,642,829.0	7.2	1 (ref.)	1 (ref.)	1 (ref.)
100–129	1,277,908	311	6,568,173.8	4.7	0.72 (0.62, 0.84)	0.72 (0.62, 0.83)	0.71 (0.61, 0.82)
130–159	807,010	178	4,147,538.5	4.3	0.66 (0.55, 0.79)	0.65 (0.54, 0.78)	0.64 (0.54, 0.77)
160–189	269,116	55	1,380,867.4	4.0	0.61 (0.46, 0.81)	0.60 (0.45, 0.79)	0.60 (0.45, 0.79)
≥190	72,319	20	369,729.7	5.4	0.83 (0.53, 1.30)	0.81 (0.51, 1.26)	0.81 (0.52, 1.27)
**TG**							
<150	2,417,850	693	12,406,271.7	5.6	1 (ref.)	1 (ref.)	1 (ref.)
150–199	520,525	132	2,673,843.1	4.9	0.84 (0.70, 1.01)	0.82 (0.68, 0.99)	0.821 (0.68, 0.99)
≥200	589,401	144	3,029,023.5	4.8	0.88 (0.73, 1.05)	0.86 (0.71, 1.03)	0.86 (0.71, 1.03)

Abbreviation: TC, total cholesterol; HDL-C, high-density lipoprotein cholesterol; LDL-C low-density lipoprotein cholesterol; TG, triglyceride; IR, incidence rate; PY, person-years; HR, hazard ratio; CI, confidence interval. Model 1 was adjusted for age and sex. Model 2 was adjusted for age, sex, body mass index, smoking, alcohol consumption, physical activity, and diabetes. Model 3 was adjusted for age, sex, body mass index, smoking, alcohol consumption, physical activity, diabetes, and lipid-lowering medication.

Table S6. STROBE checklist.

Section/Topic	Item No	Recommendation	Page/Line
Title and abstract	1	(*a*) Indicate the study's design with a commonly used term in the title or the abstract	1/44
		(*b*) Provide in the abstract an informative and balanced summary of what was done and what was found	1/41–2/60
		**Introduction**	
Background/rationale	2	Explain the scientific background and rationale for the investigation being reported	2/64–3/100
Objectives	3	State specific objectives, including any prespecified hypotheses	3/101–103
		**Methods**	
Study design	4	Present key elements of study design early in the paper	3/123
Setting	5	Describe the setting, locations, and relevant dates, including periods of recruitment, exposure, follow-up, and data collection	3/104–121, 3/128–138, 4/176–186
Participants	6	(*a*) Give the eligibility criteria, and the sources and methods of selection of participants. Describe methods of follow-up	3/128–138, 4/176–186
		(*b*) For matched studies, give matching criteria and number of exposed and unexposed	NA
Variables	7	Clearly define all outcomes, exposures, predictors, potential confounders, and effect modifiers. Give diagnostic criteria, if applicable	3/139–4/175
Data sources/ measurement	8	For each variable of interest, give sources of data and details of methods of assessment (measurement). Describe comparability of assessment methods if there is more than one group	3/128–4/175
Bias	9	Describe any efforts to address potential sources of bias	NA
Study size	10	Explain how the study size was arrived at	3/128–138
Quantitative variables	11	Explain how quantitative variables were handled in the analyses. If applicable, describe which groupings were chosen and why	5/194–197
Statistical methods	12	(*a*) Describe all statistical methods, including those used to control for confounding	5/187–206
		(*b*) Describe any methods used to examine subgroups and interactions	5/187–206
		(*c*) Explain how missing data were addressed	3/136–137
		(*d*) If applicable, explain how loss to follow-up was addressed	4/185–186
		(*e*) Describe any sensitivity analyses	4/160–161, 5/203–204
		**Results**	
Participants	13	(a) Report numbers of individuals at each stage of study—e.g., numbers potentially eligible, examined for eligibility, confirmed eligible, included in the study, completing follow-up, and analysed	NA
		(b) Give reasons for non-participation at each stage	NA
		(c) Consider use of a flow diagram	NA
Descriptive data	14	(a) Give characteristics of study participants (e.g., demographic, clinical, social) and information on exposures and potential confounders	Table 1
		(b) Indicate number of participants with missing data for each variable of interest	NA
		(c) Summarise follow-up time (e.g., average and total amount)	6/224–226
Outcome data	15	Report numbers of outcome events or summary measures over time	6/224–226
Main results	16	(*a*) Give unadjusted estimates and, if applicable, confounder-adjusted estimates and their precision (e.g., 95% confidence interval). Make clear which confounders were adjusted for and why they were included	Tables 2,3, Figure 1
		(*b*) Report category boundaries when continuous variables were categorized	NA
		(*c*) If relevant, consider translating estimates of relative risk into absolute risk for a meaningful time period	NA
Other analyses	17	Report other analyses done—e.g., analyses of subgroups and interactions, and sensitivity analyses	7/262–8/268
		**Discussion**	
Key results	18	Summarise key results with reference to study objectives	8/278–284
Limitations	19	Discuss limitations of the study, taking into account sources of potential bias or imprecision. Discuss both direction and magnitude of any potential bias	10/354–367
Interpretation	20	Give a cautious overall interpretation of results considering objectives, limitations, multiplicity of analyses, results from similar studies, and other relevant evidence	8/285–10/353
Generalisability	21	Discuss the generalisability (external validity) of the study results	10/361–365
		**Other information**	
Funding	22	Give the source of funding and the role of the funders for the present study and, if applicable, for the original study on which the present article is based	10/392

© 2021 by the authors. Licensee MDPI, Basel, Switzerland. This article is an open access article distributed under the terms and conditions of the Creative Commons Attribution (CC BY) license (http://creativecommons.org/licenses/by/4.0/).
